# Effects of the Suboccipital Muscle Inhibition Technique on the Range of Motion of the Ankle Joint and Balance According to Its Application Duration: A Randomized Controlled Trial

**DOI:** 10.3390/healthcare9060646

**Published:** 2021-05-29

**Authors:** Han-Sol Kang, Hyung-Wook Kwon, Di-gud Kim, Kwang-Rak Park, Suk-Chan Hahm, Jeong-Hyun Park

**Affiliations:** 1 Graduate School of Integrative Medicine, CHA University, Seongnam 13488, Korea; khs8407@hanmail.net; 2 Department of Anatomy & Cell Biology, Graduate School of Medicine, Kangwon National University, Chuncheon 24341, Korea; kwenhw@naver.com (H.-W.K.); oehealth@hanmial.net (D.-g.K.); airboba@naver.com (K.-R.P.)

**Keywords:** suboccipital muscle inhibition technique, ankle joint, range of motion test, lunge angle, balance, randomized controlled trial

## Abstract

This study aimed to investigate the effects of suboccipital muscle inhibition technique (SMIT) on active range of motion (AROM) of the ankle joint, lunge angle (LA), and balance in healthy adults, according to the duration of its application. A total of 80 participants were randomly allocated to the 4-min suboccipital muscle inhibition (SMI) group (SMI_4M, *n* = 20), 8-min SMI group (*n* = 20), 4-min sham-SMI (SSMI) group (*n* = 20), and 8-min SSMI group (*n* = 20). Accordingly, the SMIT and sham SMIT were applied for 4 min or 8 min in the respective groups. AROM of dorsiflexion and LA were assessed, and a single leg balance test (SLBT) was performed before and after the intervention. AROM (4 min, *p* < 0.001; 8 min, *p* < 0.001), LA (4 min, *p* < 0.001; 8 min, *p* < 0.001), and SLBT (4 min, *p* < 0.001; 8 min, *p* < 0.001) significantly improved after SMI application. Compared with the SSMI group, the SMI group showed a significant increase in AROM (*p* < 0.001), LA (*p* < 0.001), and SLBT (*p* < 0.001). Except for SLBT (*p* = 0.016), there were no significant interactions between intervention and application duration. The results suggest that the SMIT, at durations of both 4 and 8 min, could be effective tools for improving AROM, LA, and balance.

## 1. Introduction

The suboccipital muscle inhibition technique (SMIT) is associated with changes in body flexibility. Previous studies have reported that SMIT increases the range of motion (ROM) in patients with several pathologic conditions [1,2,3]. SMIT increased ROM of the hip and knee joints in patients with hamstring shorting [3], improved cervical range of motion in subjects with neck pain [1], and extension of the elbow joint in patients with cervical whiplash [2]. In addition, SMIT improved the head position of subjects with a history of orthodontia use [4] and decreased pain in subjects with a latent trigger point in the masseter muscle [5].

It is known that the suboccipital muscle has a large number of muscle spindles, which can also affect balance. According to a study by Peck et al., the rectus capitis posterior minor muscle has 36 spindles per gram, which serves as a proprioceptor, and the rectus capitis posterior major muscle has 30.5 spindles per gram [6]. Conversely, the splenius capitis muscle has 7.6 spindles per gram and only 0.8 spindles per gram for the gluteus maximus. In balance ability, muscle atrophy of the rectus capitis posterior minor muscle decreased the proprioceptive ability of the muscle [7], and the standing posture balance ability declined in the group with the rectus capitis posterior minor muscle atrophy [8]. Based on these studies, the condition of the suboccipital muscle may affect balance ability.

SMIT has been applied at various durations of intervention. Previous studies have reported a significant increase in ROM for the hip and knee joints by applying the SMIT for 2 min [3], and SMIT for 2 min significantly reduced pressure pain threshold over latent myofascial trigger points of the masticatory muscles [5]. Antolinos-Campillo et al. confirmed the increase in ROM of the elbow joint by applying SMIT for 4 min [2], Cho et al. confirmed the improvement in ROM of the hip joint by applying it for 5 min [9], and Azam reported that SMIT was applied for 8 min to confirm the improvement in the walking ability of children with cerebral palsy [10]. Despite several studies of SMIT, there are no comparisons of the effect of SMIT was investigated according to application times on ROM and balance of the ankle joint. Therefore, in this study, the effect of the SMIT according to the duration of its application on the active dorsiflexion ROM of the ankle joint, lunge angle, and balance ability in healthy adults.

## 2. Methods

### 2.1. Design and Ethics

This study was designed as a double-blind, randomized controlled trial. The study protocol was approved by the Research Ethics Committee of Kangwon National University (KWNUIRB–2019–10–001-001) and registered (WHO International Clinical Trials Registry Platform, KCT0005806). Written consent was obtained from all study participants.

### 2.2. Participants and Sample Size

The participants were healthy adults aged 30–50 years who had no problem following the researcher’s instructions. Those who had experienced acute back pain in the last six months, whiplash injuries to the cervical bone in the past, severe injuries to the ankles, and those with neuropsychiatric problems were excluded from this study’s selection process [3].

G*Power version 3.1.9.4. (Universität Kiel, Kiel, Germany) was used to calculate the number of samples. The effect size was set to 0.4 (large effect size) [11], the statistical power set to 0.9, and the significance level set to 0.05 to calculate the number of samples. On the basis of these values, 68 subjects were needed. Finally, 80 individuals participated in the study with a dropout rate of 15%.

### 2.3. Experimental Procedure

Using randomization software (https://www.randomizer.org/ accessed on 28 November 2019), the 80 participants were divided into the following groups at the same allocation ratio: SMI for 4 min (SMI_4M), SMI for 8 min (SMI_8M), sham-SMI (SSMI) for 4 min (SSMI_4M), and SSMI for 8 min (SSMI_8M). All interventions and assessments were performed at CHA University. Before and after the intervention, the active ROM (AROM) of dorsiflexion was assessed as a primary variable. Lunge angle test (LAT) and single leg balance test (SLBT) were also performed as secondary variables. Three tests were conducted again, and the means were used as representative values. They were measured by a physical therapist with more than 10 years of experience. The participant’s group information was blinded to the assessor.

### 2.4. Intervention

**Figure 1:**
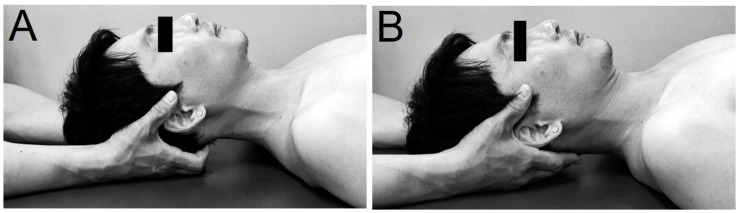
Interventions. (**A**). Suboccipital muscle inhibition technique, (**B**). Sham suboccipital muscle inhibition technique.

The SMI, which is an intervention group, was divided into SMI_4M and SMI_8M. The SMI was applied according to the method prescribed in a previous study [4] by a physical therapist with more than 10 years of experience (Figure 1A). The participant was asked to lie down facing the ceiling; the examiner sat on the head of the bed and placed his hands on the participant’s occipital bone. The examiner then used the middle and ring fingers of both his hands to find the space between the occipital bone and the atlas. Using the metacarpophalangeal joint bent at 90°, the examiner tapped the occipital bone lightly to apply constant pressure without causing pain while maintaining the 90° angle of the index, middle, and ring fingers of both hands. The examiner then gently pulled the back of the head backward to relax the muscles. Afterward, the experimenter slowly released the participant’s head and put it down slowly. The control group, where the SSMI was applied, was divided into the SSMI_4M and SSMI_8M groups, respectively. The SSMI was performed according to the method prescribed in a previous study (Figure 1B) [4]. In this technique, the examiner did not apply pressure and placed the tip of his finger under the participant’s occipital bone. The position of the finger was the same as that in the SMIT, but no force or movement was applied.

### 2.5. Outcome Measurements

#### 2.5.1. Active Dorsiflexion Range of Motion Test

AROM of dorsiflexion was performed by following the method prescribed in a previous study [12]. The participant leaned on a table to measure AROM, and the knee joint was bent at 90°. Three points were marked to measure the ankle dorsiflexion angle. The first axis was a horizontal line passing through the center point of the lateral malleolus. The second axis was a horizontal line passing through the central point of the lateral surface at the far end of the fifth metatarsal bone. The third axis was a horizontal line passing through the center point of the lateral side of the fibular head. The axis of the joint goniometer (Baseline 12-1001HR goniometer, Baseline, NY, USA) was placed on the lateral malleolus, and the fixed axis of the joint goniometer was placed horizontally at the center of the transverse lateral side of the fifth metatarsal bone. The moving axis of the joint goniometer was placed parallel to the center of the fibular head, and the angle was measured. The points marked by three dots remained at the same point until the end of the study. Before the examiner measured the AROM, the participant performed four active dorsiflexion exercises for 5 s each as a warm-up exercise. The position of the subtalar joint was controlled by the examiner’s hand, and the examiner measured the ankle joint angle three times in the maximum AROM. The average values of the left and right sides were used as the values of each participant’s AROM. The standard error of measurement and minimal detectable change for the goniometer was 1.8–2.8° and 5.0–7.7°, respectively [13].

#### 2.5.2. Lunge Angle Test

LAT was performed according to the method prescribed by Alon et al. [14]. The examiner instructed the participant to stand with the heel and second toe on the straight line. The second toe was placed at a distance of 50 cm from the wall, and when the participant performed a lunge movement forward, the knee was pushed as close to the wall as possible without lifting the heel. The examiner confirmed that each participant’s heel was always in contact with the floor during the measurement. The direction of the knee joint movement of the additionally tested when the foot was moved forward and aligned above the second toe through minimal pronation. A gravimetric inclinometer (Baseline Inclinometer, White Plains, NY, USA) was used to measure the tibial angle in the lunge position. The gravimetric inclinometer was placed 15 cm below the tuberosity of the tibia. The tibial tuberosity of the tested foot was marked with a pen for consistent placement of the inclinometer. The lunge angle was measured three times at the endpoint in front of the tibia. The average values of the left and right sides were used as the lunge angle values for each participant. The standard error of measurement and minimal detectable change for the goniometer was 1.3–1.4° and 3.7–3.8°, respectively [13].

#### 2.5.3. Single Leg Balance Test

An SLBT was performed to quantify balance ability [15]. The examiner visually checked the participant’s previous ankle injury condition. In this test, the participants were asked to remove their shoes, stand with one leg straight, and the other leg bent at the hip and knee joint to 90°, and not touch the leg supporting the weight. Participants were asked to fix their gaze at the point marked on the wall and put their hands on their sides for balance. A stopwatch was used to record the time (in seconds) until the participant lost balance, fell off his/her hand, or put his/her foot on the floor; then, both legs were examined. The average values of the left and right sides were used as the single leg balance values of each participant.

### 2.6. Data Analysis

Data analysis was performed using SPSS version 21.0 (IBM SPSS, Chicago, IL, USA), and the measured values of all variables were calculated as mean, standard deviation, or the number of participants. The Kolmogorov-Smirnov test was performed to investigate if each variable was likely to follow a normal distribution, and the normality was assessed for all dependent variables. The general characteristics of the participants between groups were confirmed using a chi-squared test or a one-way analysis of variance (ANOVA). Changes in the measured variables within the group were analyzed using a paired *t*-test. Two-way ANOVA was used to confirm the differences in intervention, application time, and interaction between intervention and application time in AROM, LAT, and SLBT, respectively. The statistical significance level was set at α = 0.05.

## 3. Results

### 3.1. Characteristics of Participants

**Figure 2:**
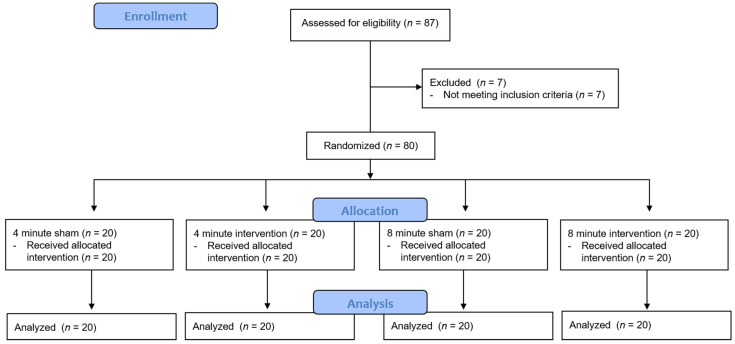
Flow diagram of the participants.

Eighty-seven participants volunteered for the experiment, and seven were excluded as they did not meet the inclusion criteria. Finally, 80 participants who met the inclusion criteria participated in the study (Figure 2).

There were no differences among the four groups in terms of sex, age, height, and weight (Table 1).

### 3.2. Changes in AROM of Dorsiflexion

In AROM, SMI_4M significantly increased after intervention (*t* = −14.875, *p* < 0.001), and SMI_8M also significantly improved after the intervention (*t* = −9.752, *p* < 0.001) (Table 2).

However, neither SSMI_4M nor SSMI_8M showed significant improvement in AROM. In the case of SMI and SSMI, there was a significant difference between interventions in AROM (*F* = 237.613, *p* < 0.001, Partial *η*^2^ = 0.758) (Table 3). There was no significant difference in AROM according to the duration of application (Table 3). There was no significant interaction between the application method and application duration in AROM (Table 3).

### 3.3. Changes in Lunge Angle

At lunge angle, SMI_4M significantly increased after intervention (*t* = −12.177, *p* < 0.001). SMI_8M also significantly improved after the intervention (*t* = −12.575, *p* < 0.001) (Table 2). However, neither SSMI_4M nor SSMI_8M showed significant improvement in the lunge angle. In the case of SMI and SSMI, there was a significant difference between interventions in lunge angle (*F* = 261.920, *p* < 0.001, Partial *η*^2^ = 0.775) (Table 3). There was no significant difference in the lunge angle according to the application duration (Table 3). There was no significant interaction between the application method and intervention time in lunge angle (Table 3).

### 3.4. Changes in Balance Ability

In balance ability, SMI_4M significantly increased after intervention (*t* = −4.647, *p* < 0.001), and SMI_8M also significantly improved after the intervention (*t* = −6.761, *p* < 0.001) (Table 2). However, neither SSMI_4M nor SSMI_8M showed significant improvement in balance ability. In the case of SMI and SSMI, there was a significant difference between interventions in balance ability (*F* = 45.764, *p* < 0.001, Partial *η*^2^ = 0.376) (Table 3). There was no significant difference in lunge angle according to application time (Table 3). A significant interaction between the application method and intervention time in lunge angle was observed (*F* = 45.764, *p* = 0.016, Partial *η*^2^ = 0.074) (Table 3).

## 4. Discussion

This study aimed to investigate the effect of SMIT according to the duration of its application on AROM, lunge angle, and balance ability in healthy adults. This study is the first investigation to show that both SMI for 4 min and 8 min could improve AROM, lunge angle, and balance ability. These results may provide evidence for the use of SMIT for the improvement of motor function.

In this study, a significant increase in AROM of the ankle joint was confirmed after SMI compared with SSMI. Grieve et al. reported an increase in the ability to lean forward to the upper body after applying the self-fascial release technique to the plantar tendon [16]. In addition, in previous studies, it was confirmed that SMI increased the flexibility of the hip and knee joints [3,9], and suboccipital stretching application increased hip flexion ROM [17]. Considering that the results of previous studies support the results of this study and the plantar fascia, gastrocnemius, soleus, hamstrings, sacrotuberous ligament, erector spinae, suboccipital muscle, and epicranial fascia are connected by the superficial back line [18], and the suboccipital muscle connects the fascia of the lower limb, SMI may affect the increase of ROM of the ankle joint.

The decreased tension in nerves that innervate the dorsiflexors by SMI may also be the cause of the increased ROM of the ankle joint. Hack et al. reported the myodural bridge that connects the rectus capitis posterior minor muscle (RCPM), one of the suboccipital muscles, and posterior atlanto-occipital membrane [19]. The increase of distal ROM by manual application applying to the suboccipital muscle may be related to the decrease of peripheral nerve tension connected to the myodural bridge [17]. A previous study [2] that reported the increased extension of the elbow joint by the decreased tension in the median nerve following SMI may support our result in this study. Taken together, SMI applied to suboccipital muscles may result in decreased peripheral nerve tension by reduction of dural tension of the myodural bridge, which may increase the ROM of the ankle.

In previous studies, the intervention time of SMI was applied under various conditions. Aparicio et al. reported an improvement in ROM of the hip and knee joints with 2 min of intervention [3], and Lee et al. reported a reduction of headache and improvement of neck function with 3 min of intervention [20] Antolinos-Campillo et al. demonstrated an increase in hip joint ROM with 5 min of intervention [2] and Azam reported that an 8 min intervention improved the gait function of children with cerebral palsy [10]. In this study, there were no significant differences in AROM, lunge angle, and balance ability according to the application time (4 min vs. 8 min). In previous studies, it was suggested to maintain the SMI between 2.5 and 5 min to increase the flexibility of the fascia [21]. Based on this, it is considered that a significant effect was confirmed even in the application of SMI for 4 min in this study.

SMIT significantly increased balance ability. A previous study reported a significant increase in walking ability by an increase in calf and hamstring elasticity following SMI, which may be related to the presence of a myodural bridge connecting RCPM to the duramater, in 30 children with cerebral palsy [10]. In addition, Mecagni et al. suggested that there is a correlation between the increase of ROM and the improvement of balance ability [22]. Thus, increased ROM by SMI in this study may affect balance ability. These results suggest that inhibition of the suboccipital muscles improved balance ability. In this study, 8 min of intervention time was more effective than 4 min in balance ability. However, the basis for the significant interaction according to the application time of SMI on balance ability is not yet clear and requires additional research.

This study has some limitations. First, the duration of the effect cannot be predicted by confirming the immediate effect of the intervention by performing a one-time SMI and measuring it immediately. Further studies, including follow-up tests, should be conducted. Second, since the effect on long-term and repeated application of SMI has not been assessed, it is necessary to confirm it through further studies. Third, since the results were limited to healthy adults, it is necessary to confirm the efficacy of SMIT in a variety of patients.

## 5. Conclusions

The SMIT at durations of both 4 and 8 min improved AROM of the ankle, lunge angle, and balance ability. Thus, applying SMI for short periods may help improve motor function.

## Figures and Tables

**Table 1:** General characteristics of participants by study group.

Variables	SMI_4M (*n* = 20)	SSMI_4M (*n* = 20)	SMI_8M (*n* = 20)	SSMI_8M (*n* = 20)	*χ* ^2^ *or F*	*p*
Sex^†^						
Male	11	11	11	11	0.000	1.000
Female	9	9	9	9		
Age (year)	37.7 ± 7.3	37.5 ± 6.4	37.5 ± 5.3	37.7 ± 5.3	0.007	0.999
Height (cm)	171.3 ± 8.8	171.6 ± 7.2	171.5 ± 9.2	171.3 ± 9.7	0.007	0.999
Weight (kg)	68.6 ± 13.8	67.7 ± 14.3	68.2 ± 15.1	68.1 ± 13.8	0.012	0.998

SMI_4M, suboccipital muscle inhibition for 4 min; SSMI_4M, sham suboccipital muscle inhibition for 4 min; SMI_8M, suboccipital muscle inhibition for 8 min; SSMI_8M, sham suboccipital muscle inhibition for 8 min. Values are expressed as mean ± SD or ^†^ number of participants.

**Table 2:** Changes of AROM, LA, and balance in each group.

Variables	Pre	Post	*t*	*p*	*d*
AROM (°)					
SMI_4M (*n* = 20)	23.0 ± 5.1	29.5 ± 5.6	−14.875	<0.001 *	3.326
SSMI_4M (*n* = 20)	25.3 ± 4.9	25.6 ± 4.9	−1.312	0.205	0.293
SMI_8M (*n* = 20)	25.0 ± 4.1	30.6 ± 5.2	−9.752	<0.001 *	2.181
SSMI_8M (*n* = 20)	25.0 ± 5.8	25.2 ± 5.8	−1.291	0.212	0.289
LA (°)					
SMI_4M (*n* = 20)	46.1 ± 6.9	51.2 ± 6.7	−12.177	<0.001 *	2.723
SSMI_4M (*n* = 20)	47.4 ± 5.6	47.2 ± 5.9	1.080	0.294	0.241.
SMI_8M (*n* = 20)	48.4 ± 5.2	52.6 ± 5.8	−12.575	<0.001 *	2.812
SSMI_8M (*n* = 20)	48.4 ± 5.2	52.6 ± 5.8	−0.839	0.412	0.188
Balance (sec)					
SMI_4M (*n* = 20)	81.7 ± 31.1	109.9 ± 42.6	−4.647	<0.001 *	1.039
SSMI_4M (*n* = 20)	80.1 ± 45.4	86.3 ± 44.5	−1.841	0.081	0.412
SMI_8M (*n* = 20)	72.7 ± 33.4	119.5 ± 45.3	−6.761	<0.001 *	1.512
SSMI_8M (*n* = 20)	91.8 ± 34.5	91.6 ± 35.2	0.080	0.937	0.018

AROM, active dorsiflexion range of motion; LA, lunge angle; SMI_4M, suboccipital muscle inhibition for 4 min; SSMI_4M, sham suboccipital muscle inhibition for 4 min; SMI_8M, suboccipital muscle inhibition for 8 min; SSMI_8M, sham suboccipital muscle inhibition for 8 min; *d*: Effect size caculated with Cohen’s d. Values are expressed as mean ± SD. * *p* < 0.05.

**Table 3:** Comparisons in AROM, LA, and SLBT for the intervention and application duration.

Variables	SMI_4M (*n* = 20)	SSMI_4M (*n* = 20)	SMI_8M (*n* = 20)	SSMI_8M (*n* = 20)		*F*	*p*	Partial *η^2^*	*R^2^*
AROM (°)	6.5 ± 1.9	0.2 ± 0.7	5.6 ± 2.6	0.2 ± 0.8	I	237.613	<0.001 *	0.758	0.762
					T	1.421	0.237	0.018	
					I×T	1.358	0.248	0.018	
LA (°)	5.1 ± 1.9	−0.2 ± 0.8	4.3 ± 1.5	0.1 ± 0.7	I	261.920	<0.001 *	0.775	0.778
					T	0.829	0.365	0.011	
					I×T	3.637	0.060	0.046	
Balance (sec)	28.1 ± 27.1	6.2 ± 15.1	46.8 ± 30.9	−0.2 ± 12.6	I	45.764	<0.001 *	0.376	0.412
					T	1.440	0.234	0.019	
					I×T	6.057	0.016 *	0.074	

AROM, active dorsiflexion range of motion; LA, lunge angle; SMI_4M, suboccipital muscle inhibition for 4 min; SSMI_4M, sham suboccipital muscle inhibition for 4 min; SMI_8M, suboccipital muscle inhibition for 8 min; SSMI_8M, sham suboccipital muscle inhibition for 8 min; I, intervention (SMI vs. SSMI); T, application duration (4 min vs. 8 min); T×I, application time and duration interaction; η^2^, eta squared; R^2^, coefficient of variation. Values are expressed as mean ± SD in post values–pre values. * *p* < 0.05.

## Data Availability

The data presented in this study are available on request from the corresponding author.
